# Engineering of *Aeromonas caviae* Polyhydroxyalkanoate Synthase Through Site-Directed Mutagenesis for Enhanced Polymerization of the 3-Hydroxyhexanoate Unit

**DOI:** 10.3389/fbioe.2021.627082

**Published:** 2021-03-03

**Authors:** Ken Harada, Shingo Kobayashi, Kanji Oshima, Shinichi Yoshida, Takeharu Tsuge, Shunsuke Sato

**Affiliations:** ^1^Department of Materials Science and Engineering, Tokyo Institute of Technology, Yokohama, Japan; ^2^Biotechnology Research Laboratories, Kaneka Corporation, Hyogo, Japan

**Keywords:** polyhydroxyalkanoate, PHA synthase, copolymer composition, site-directed mutagenesis, homology modeling

## Abstract

Polyhydroxyalkanoate (PHA) synthase is an enzyme that polymerizes the acyl group of hydroxyacyl-coenzyme A (CoA) substrates. *Aeromonas caviae* PHA synthase (PhaC_Ac_) is an important biocatalyst for the synthesis of a useful PHA copolymer, poly[(*R*)-3-hydroxybutyrate-*co*-(*R*)-3-hydroxyhexanoate] [P(3HB-*co*-3HHx)]. Previously, a PhaC_Ac_ mutant with double mutations in asparagine 149 (replaced by serine [N149S]) and aspartate 171 (replaced by glycine [D171G]) was generated to synthesize a 3HHx-rich P(3HB-*co*-3HHx) and was named PhaC_Ac_ NSDG. In this study, to further increase the 3HHx fraction in biosynthesized PHA, PhaC_Ac_ was engineered based on the three-dimensional structural information of PHA synthases. First, a homology model of PhaC_Ac_ was built to target the residues for site-directed mutagenesis. Three residues, namely tyrosine 318 (Y318), serine 389 (S389), and leucine 436 (L436), were predicted to be involved in substrate recognition by PhaC_Ac_. These PhaC_Ac_ NSDG residues were replaced with other amino acids, and the resulting triple mutants were expressed in the engineered strain of *Ralstonia eutropha* for application in PHA biosynthesis from palm kernel oil. The S389T mutation allowed the synthesis of P(3HB-*co*-3HHx) with an increased 3HHx fraction without a significant reduction in PHA yield. Thus, a new workhorse enzyme was successfully engineered for the biosynthesis of a higher 3HHx-fraction polymer.

## Introduction

Polyhydroxyalkanoates (PHAs) are bio-based polyesters produced by a wide range of microorganisms as carbon and energy storage materials. The wild-type strain H16 of *Ralstonia eutropha* (or *Cupriavidus necator*) is one of the best-known PHA-producing bacteria (Sudesh et al., 2000; Steinbüchel and Hein, 2001). There has been long-standing interest in using PHAs as biodegradable bioplastics that could serve as alternatives to petrochemical plastics. Recently, PHAs have attracted attention as biodegradable and biocompatible thermoplastics for use in a wide range of agricultural, marine, and medical applications because of their excellent biodegradability (Akiyama et al., 2003).

Polyhydroxyalkanoates mainly consist of short-chain length (SCL; C3 to C5) and/or medium-chain-length (MCL; C6 and longer) monomers (Rehm, 2003). Among the SCL-PHAs, poly[(*R*)-3-hydroxybutyrate] [P(3HB)] is the most common bacterial PHA in nature. Although P(3HB) is a highly crystalline, hard, and brittle polymer, it begins to decompose at a temperature close to its melting point, making it difficult to process this polymer (Lehrle and Williams, 1994). Copolymerization of MCL monomers with a 3HB unit leads to notable changes in the physical properties of PHA, depending on the molecular structure and copolymer composition (Noda et al., 2005). The best-studied SCL/MCL-PHA copolymer is poly(3HB-*co*-3-hydroxyhexanoate) [P(3HB-*co*-3HHx)]. In this polymer, an important aspect is to control the level of the 3HHx monomer fraction for practical application in many fields. For example, the elongation at break increases from 5 to 760% by increasing the 3HHx fraction from 0 to 15 mol% (Doi et al., 1995; Chen et al., 2000; Andreeßen et al., 2014). P(3HB-*co*-3HHx) with 10–15 mol% 3HHx fraction can be used as an alternative to conventional plastics such as polypropylene and polyethylene (Shimamura et al., 1994; Chen et al., 2000; Andreeßen et al., 2014). However, it is difficult to efficiently produce P(3HB-*co*-3HHx) with such a high 3HHx fraction. Thus, significant efforts have been made to increase the 3HHx fraction in P(3HB-*co*-3HHx) biosynthesis (Jian et al., 2010; Budde et al., 2011; Arikawa and Matsumoto, 2016a).

The bacterium *Aeromonas caviae* is an original strain that can produce P(3HB-*co*-3HHx) from plant oils and fatty acids (Shimamura et al., 1994). *Aeromonas caviae* PHA synthase (PhaC_Ac_) shows substrate specificity toward 3HB and 3-hydroxyvalerate monomers, as well as the 3HHx monomer (Kobayashi et al., 1994). From this point of view, PhaC_Ac_ is a valuable biocatalyst for production of P(3HB-*co*-3HHx). However, the polymer production capacity of *A. caviae* is not superior to that of other PHA producers. With the help of genetic engineering, recombinant *R. eutropha* expressing PhaC_Ac_ was generated, which demonstrated remarkable enhancement of P(3HB-*co*-3HHx) production from plant oils (Fukui and Doi, 1997, 1998; Kahar et al., 2004).

Additionally, to increase the 3HHx fraction in P(3HB-*co*-3HHx), various strategies have been developed. One effective approach is to increase the expression of (*R*)-specific enoyl-coenzyme A (CoA) hydratase (PhaJ4b_Re_), which provides *R*-3-hydroxyacyl-CoA precursors for PHA synthesis from the β-oxidation cycle, to reinforce the supply of the 3HHx monomer (Arikawa and Matsumoto, 2016a). In contrast, the 3HHx fraction in the polymer was increased by deleting the gene for the 3HB supplier acetoacetyl-CoA reductase (PhaB_Re_) to suppress the 3HB monomer supply; however, the PHA yield decreased (Budde et al., 2011).

Another approach to increase the 3HHx fraction in PHA is the engineering of PHA synthase (Kichise et al., 2002; Tsuge et al., 2004, 2007a,b; Watanabe et al., 2012). In previous studies, PhaC_Ac_ was modified via evolutionary engineering approaches, and several mutation sites (e.g., asparagine 149, aspartate 171, valine 214, and phenylalanine 518) enhanced the 3HHx polymerization capacity (Amara et al., 2002; Kichise et al., 2002; Tsuge et al., 2004). Furthermore, a double mutant of PhaC_Ac_, termed the NSDG mutant, which has two amino acid substitutions of asparagine 149 by serine (N149S) and aspartate 171 by glycine (D171G), was generated as a superior enzyme capable of synthesizing P(3HB-*co*-3HHx) with a higher 3HHx fraction than the wild-type enzyme (Tsuge et al., 2007b). However, since then, no PhaC_Ac_ mutant with further high 3HHx polymerization ability has been created.

The three-dimensional structure of a protein provides important information for understanding its biochemical function and catalytic mechanism. Homology modeling aims to build three-dimensional protein structure models using experimentally determined structures of related family members as templates. Thus, homology modeling is a powerful tool for understanding and predicting the three-dimensional structure of unknown proteins to determine beneficial mutation sites and improve protein properties (Stoilov et al., 1998; Lee et al., 2011). Recently, some research groups have determined the partial crystal structure of *R. eutropha* PHA synthase (PhaC_Re_), which is classified into the same group (class I) as PhaC_Ac_ based on its substrate specificity and subunit structure (Wittenborn et al., 2016; Kim et al., 2017). According to their crystal structures, three active residues, Cys319, Asp480, and His508, in PhaC_Re_ are in close proximity. Additionally, amino acid residues that make up the substrate pocket have been identified (Wittenborn et al., 2016; Kim et al., 2017). Moreover, structural information on the available PHA synthases has been increasing (Chek et al., 2017, 2019, 2020).

In this study, using a newly constructed homology model of PhaC_Ac_, three amino acid residues were predicted to be constituents of the substrate pocket and involved in substrate recognition. Based on this prediction, site-specific mutagenesis was conducted on PhaC_Ac_ NSDG to introduce an additional third mutation. The resulting triple mutants were expressed in the strain 005dC1Z126TRCB, an engineered *R. eutropha* strain, grown on palm kernel oil as a carbon source for PHA biosynthesis. It was found that the triple mutant PhaC_Ac_ NSDG/S389T is capable of synthesizing P(3HB-*co*-3HHx) with a higher 3HHx fraction than the parental PhaC_Ac_ NSDG. Furthermore, the selected PhaC_Ac_ triple mutants were isolated as PHA granule-associated enzymes from *R. eutropha* and characterized through enzyme kinetic analysis to understand how the catalytic function changed.

## Materials and Methods

### Bacterial Strains and Plasmids

Bacterial strains and gene expression plasmids used are listed in Table 1. All *Escherichia coli* strains were grown in Luria-Bertani (LB) medium. The *E. coli* strains JM109 and S17-1 were used for plasmid construction and as donors in the intergeneric conjugation experiments, respectively. All *R. eutropha* strains were grown in nutrient broth (Difco Laboratories, Detroit, MI, United States).

**TABLE 1:** Bacterial strains and gene expression plasmids.

**Strain or plasmid**	**Description**	**References**
***Ralstonia eutropha***		
H16	Wild type	ATCC17699
CnTRCB	H16 derivative; Δ*phaC_Re_::phaC_Ac_NSDG*, Δ*phaZ1*,Δ*phaZ2*,Δ*phaZ6*, P_trc_-*phaJ4b*_Re_	Arikawa and Matsumoto, 2016a
005dC1Z126TRCB	CnTRCB derivative; Δ*phaC*	This study
***Escherichia coli***		
JM109	*recA1 endA1 gyrA96 thi hsdR17 supE44 relA1*Δ*(lac-proAB)/F’ [traD36 proAB^+^ lacI^q^ lacZ*Δ*M15]*	Takara Bio.
S17-1	*recA pro hsdR RP4-2-Tc::Mu-Km::Tn7*	ATCC47055
***Plasmid***		
pNS2X-sacB	Suicide vector; Km^r^	Sato et al., 2013
pNS2X-sacB-phaC1AdS	Deletion vector for *phaC*, fragments up- and downstream of *phaC* cloned between *Swa*I site of pNS2X-sacB	Sato et al., 2013
pCUP3	Stable plasmid vector in *R. eutropha*, Km^r^	Sato et al., 2013
pCUP3-P_trp_-WT-PhaCAc	P_trp_-WT-PhaC_Ac_ expression cassette cloned into pCUP3	This study
pCUP3-P_trp_-NSDG	P_trp_-PhaC_Ac_NSDG mutant expression cassette cloned into pCUP3	This study
pCUP3-P_trp_-NSDG-Y318X	P_trp_-PhaC_Ac_NSDG-Y318X mutant expression cassette cloned into pCUP3	This study
pCUP3-P_trp_-NSDG-S389X	P_trp_-PhaC_Ac_NSDG-S389X mutant expression cassette cloned into pCUP3	This study
pCUP3-P_trp_-NSDG-L436X	P_trp_-PhaC_Ac_NSDG-L436X mutant expression cassette cloned into pCUP3	This study

To delete the *phaC_Ac_NSDG* gene in the *R. eutropha* CnTRCB strain (Arikawa and Matsumoto, 2016a), the gene deletion plasmid pNS2X-sacB-phaC1AdS (Sato et al., 2013) was introduced into the CnTRCB strain by conjugation from the donor strain *E. coli* S17-1. The deletion of *phaC* was confirmed through PCR. The resulting strain was named 005dC1Z126TRCB, which retained *phaA* and *phaB* involved in the 3HB monomer supply and provided greater proportions of 3HHx than the H16 strain, by enhancing the expression of *phaJ4b*_Re_.

### Homology Modeling of *A. caviae* PHA Synthase

A template-based modeling method using HyperChem (HYPERCUBE, INC., Gainsville, FL, United States) (Froimowitz, 1993) was used to predict the structure of PhaC_Ac_ using PDB:5T6O (residues 201–589) from PhaC_Re_ as a template.

### Plasmid Construction and Site-Directed Mutagenesis

Plasmids expressing wild-type PhaC_Ac_, the double mutant NSDG, and the triple mutants NSDG-Y318/S389/L436X were constructed based on the pCUP3 vector, which is stably maintained in *R. eutropha* (Sato et al., 2013). The wild-type *pha*C_Ac_ (*WT-phaC_Ac_*) and *phaC_Ac_ NSDG* genes were obtained through PCR with MunI_PhaCAc_F and SpeI_PhaCAc_R as primers, using the plasmid pColdI::*phaC*_Ac_ and the genomic DNA of the *R. eutropha* strain KNK005 as a template, respectively (Sato et al., 2013; Ushimaru et al., 2014). These fragments were digested by *Mun*I and *Spe*I, and then cloned into the same sites of the pCUP3 vector. The P_trp_ fragment, which was amplified by PCR using pKK388-1 (Clontech, Palo Alto, CA, United States) as a template (Arikawa and Matsumoto, 2016a), was digested with *Mun*I and ligated with *Mun*I*-*digested pCUP3 vectors carrying *WT-phaC_Ac_* and *phaC_Ac_ NSDG* genes to yield pCUP3-P_trp_-*WT-phaC_Ac_* and pCUP3-P_trp_-*phaC_Ac_ NSDG*, respectively. Site-directed mutagenesis of *phaC_Ac_NSDG* gene was performed by overlap extension PCR (Ho et al., 1989). Reverse primers containing a point mutation were designed as listed in Supplementary Table S1, and primers containing a restriction enzyme site were designed as (pCUP3_IF_MunI_trp_F) 5′-**ACA TTGCGCTGAAAGAAGGGC**CAATTGTGCTTCTGGCGTC-3′ and (pCUP3_SpeI_IF_R) 5′-**GCTCGGATCC**ACTAGTCGGCT GCCGACTGGT-3′ (the underlined sequences indicate the *Mun*I and *Spe*I sites, and the bold sequences indicate in-fusion alignment). Using the corresponding primers in phaC_Ac__Y318/S389/L436X_R and phaC_Ac__Y318/S389/L436X_F (Supplementary Table S1), the DNA fragments were amplified. The resulting fragments after one round of PCR were used as templates, and PCR was performed again using the outside primers with *Mun*I and *Spe*I sites. The resulting *phaC_Ac_NSDG* fragments with point mutations were digested using *Mun*I and *Spe*I, and then inserted into the corresponding restriction sites in the pCUP3 vector. The resulting pCUP3-P_trp_-NSDG-Y318/S389/L436X plasmids were introduced into an engineered strain of *R. eutropha* 005dC1Z126TRCB strain, in which *phaC* gene was disrupted. Transformation was performed through electroporation, as described previously (Sato et al., 2013; Arikawa et al., 2016b).

### PHA Accumulation From Palm Kernel Oil

Polyhydroxyalkanoate production was performed in 50 mL of mineral salt (MS) medium (Kato et al., 1996) with 1.29 g/L (NH_4_)_2_SO_4_ and 1.5 w/v% palm kernel oil as a sole carbon source for 72 h. Kanamycin was added to the medium at a concentration of 50 mg/L to maintain the plasmid in the cells. After cultivation, the collected cells were washed with water and ethanol to remove the remaining carbon sources and then lyophilized (Arikawa et al., 2016b). The PHA content in the cells was determined by gas chromatography (GC) after methanolysis of approximately 15 mg of lyophilized cells in the presence of 15% (v/v) sulfuric acid, as previously described (Lakshman and Shamala, 2006).

### Kinetic Analysis of the Granule-Associated PHA Synthase

The PHA synthase activity assay was performed, wherein the amount of CoA released was measured using 5,5-dithiobis(2-nitrobenzoic acid) (DTNB) with the following modifications: PHA synthase assay was initiated by adding the granule-associated PhaC_Ac_, which was obtained from 24 h of *R. eutropha* culture broth by ultracentrifugation as previously described (Valentin and Steinbüchel, 1994; Harada et al., 2019). After incubation with the substrate, *R*-3HB-CoA (0.1–20 mM) or *R*-3HHx-CoA (0.1–20 mM) (synthesized by China Suli Co., Ltd., China) at 30°C for 30 s, the reaction was stopped by adding trichloroacetic acid (2.5% (v/v) final concentration). A portion of the reaction mixture was diluted with DTNB solution (10 mM DTNB in 500 mM potassium phosphate buffer, pH 8.0) to obtain a final concentration of 1 mM DTNB. The reaction mixtures were incubated for 30 min in the dark and then centrifuged. The absorbance of the supernatant was measured at 412 nm. The molar absorbance coefficient ε_412_ = 13,600 M^–1^cm^–1^ was used to determine the concentration of the thiol group of free CoA.

### Analysis of the PHA Synthase Concentration Through Western Blotting

The concentration of the granule-associated PhaC_Ac_ was determined as previously described (Harada et al., 2019), after incubation with rabbit antiserum against a peptide from the C-terminus of PhaC_Ac_, followed by incubation with a goat anti-rabbit antibody conjugated with horseradish peroxidase (HRP; Santa Cruz Biotechnology, CA, United States). Proteins were visualized using the ECL Plus Western Blotting Detection Reagent (Bio-Rad, Hercules, CA, United States). Data were recorded using a CCD camera FAS-1000 (Toyobo, Osaka, Japan). Quantitative analysis of PhaC_Ac_ concentration on PHA granules was performed using calibration curves prepared using purified PhaC_Ac_ (130–520 ng). Band intensities were quantified using the ImageJ software^1^.

## Results

### Amino Acid Residues That Determine the Substrate Pocket Size of PhaC_Ac_

**FIGURE 1:**
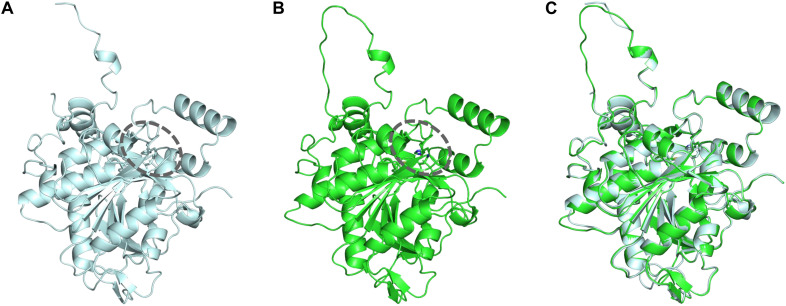
**(A)** Crystal structure of the PhaC_Re_ catalytic domain (amino acid sequence 201–589). **(B)** Homology model of PhaC_Ac_ catalytic domain (amino acid sequence 201–581). **(C)** Overlay of PhaC_Re_ (light blue) with the homology model PhaC_Ac_ (green). Dotted circle indicates the substrate pocket.

**FIGURE 2:**
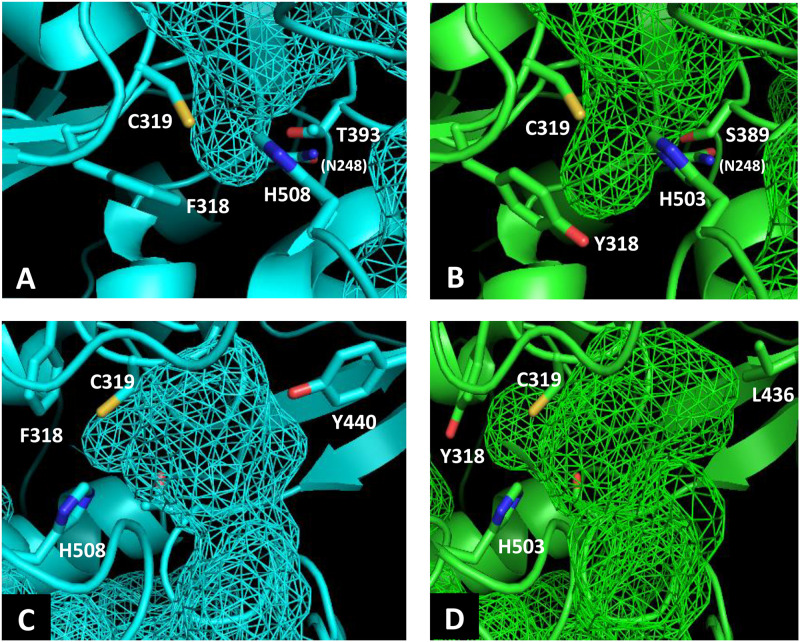
Comparison of the substrate pocket around the active site between PhaC_Re_ **(A)** and PhaC_Ac_ **(B)**. Comparison of the substrate entrance tunnel between **(C)** PhaC_Re_ and **(D)** PhaC_Ac_. Cavity spaces are indicated with a mesh.

To identify the beneficial mutation site for increasing the 3HHx fraction, a homology model of PhaC_Ac_ was first built by targeting PhaC_Re_. The PhaC_Re_ template had 38.3% (220/575) sequence identity and 75.7% (435/575) similarity with PhaC_Ac_, while in residues 201–589, sequence identity was 43.2% (164/380) and similarity was 81.8% (311/380). The constructed homology model is shown in Figures 1 and 2. Compared to the structure of PhaC_Re_, PhaC_Ac_ has a partly wide and deep cavity space around the catalytic domain (Figures 2A,B). This is in good agreement with the experimental observation that PhaC_Ac_ has a broader substrate specificity than PhaC_Re_ (Fukui and Doi, 1997). From the comparison of these structural models, two amino acid residues adjacent to the active center (PhaC_Ac_ vs. PhaC_Re_: Y318 vs. F318, S389 vs. T393) were found to be different. It was presumed that Y318 and S389 determine the depth and width of the substrate pocket of PhaC_Ac_, respectively. The substrate entrance tunnel of these models was further compared (Figures 2C,D), and additional differences were found (PhaC_Ac_ vs. PhaC_Re_: L436 vs. Y440). In PhaC_Ac_, L436 mainly contributes to expanding the substrate entrance tunnel, because there is a significant difference in the amino acid size at the homologous positions in these structural models.

### PHA Synthesis by PhaC_Ac_ NSDG With an Additional Y318 Mutation

As the Y318 of PhaC_Ac_ was predicted to determine the depth of the substrate pocket based on the homology model, we investigated the effect of the amino acid size at this position on 3HHx polymerization ability. To replace Y318, we selected Leu, Ile, and Met, which are smaller than Tyr, with the aim of expanding the substrate pocket space. The three PhaC_Ac_ mutants with NSDG mutations and either Y318L/I/M mutations were generated by site-directed mutagenesis and expressed in the engineered *R. eutropha* strain 005dC1Z126TRCB to induce P(3HB-*co*-3HHx) biosynthesis from palm kernel oil. The results are presented in Table 2. The strain expressing the wild-type enzyme accumulated 80.3 wt% P(3HB-*co*-3HHx) of dried cells, with 7.4 mol% of 3HHx fraction. Meanwhile, the strain expressing PhaC_Ac_ NSDG accumulated 85.7 wt% P(3HB-*co*-3HHx) of dried cells with 13.1 mol% of 3HHx fraction. A very small amount (less than 0.1 mol%) of 3-hydroxyoctanoate (3HO) was also detected, which is consistent with previous study (Tsuge et al., 2007b). PhaC_Ac_ NSDG was confirmed to have the ability to synthesize P(3HB-*co*-3HHx) with a higher 3HHx fraction than the wild-type enzyme. Compared to NSDG and NSDG/Y318X, a slight increase in the 3HHx fraction was observed in the strain expressing the NSDG/Y318I mutant, whereas the other two strains showed a considerable decrease in the 3HHx fraction. As for the NSDG/Y318L mutant, it showed a slight increase (0.8 mol%) in the 3HO fraction. On the contrary, expression of the NSDG/Y318I mutant notably decreased polymer accumulation (11.7 wt%) in the cells compared to the parental NSDG strain (85.7 wt%). Thus, additional mutagenesis of Y318 was not beneficial.

**TABLE 2:** Polyhydroxyalkanoate accumulation in *Ralstonia eutropha* strain 005dC1Z126TRCB by expressing PhaC_Ac_ NSDG with Y318X mutation.

**Expressed PhaC_Ac_**	**Dry cell wt. (g/L)**	**PHA content (wt.%)**	**PHA concentration (g/L)**	**Residual cell mass (g/L)**	**3HHx (mol%)**	**3HO (mol%)**	**Size increment (Å^3^)^a^**
Wild type	17.2 ± 0.4	80.3 ± 1.2	13.8 ± 0.4	3.4 ± 0.2	7.4 ± 0.1	0	-
NSDG	17.8 ± 0.3	85.7 ± 0.8	15.2 ± 0.3	2.5 ± 0.1	13.1 ± 0.1	trace	0
NSDG/Y318L	15.2 ± 0.2	77.1 ± 3.2	11.7 ± 0.4	3.5 ± 0.7	4.7 ± 0.1	0.8 ± 0.1	−17
NSDG/Y318I	2.5 ± 0.3	11.7 ± 1.3	0.3 ± 0.0	2.2 ± 0.3	13.9 ± 0.7	0	−17
NSDG/Y318M	15.5 ± 0.2	85.2 ± 1.4	13.2 ± 0.4	2.3 ± 0.8	2.1 ± 0.5	trace	−17

*Cells were cultured in mineral salt medium containing 1.5 w/v% palm kernel oil at 30 °C for 72 h.*

*Results are mean ± standard deviations from three separate experiments.*

*3HHx: 3-hydroxyhexanoate, 3HO: 3-hydroxyoctanoate, PHA: Polyhydroxyalkanoate, trace: less than 0.1 mol%.*

*^a^Size increment at position 318 from the wild-type PhaC_Ac_.*

### PHA Synthesis by PhaC_Ac_ NSDG With an Additional S389 Mutation

S389 in PhaC_Ac_ contributes to cavity formation near the active center. It is homologous to T393 in PhaC_Re_, and the cavity space in PhaC_Ac_ is larger due to the volume of one methyl group. To further expand the cavity space, the amino acid residue at position 389 was replaced with Ala (S389A), which is a smaller amino acid. To examine the opposite effect on the amino acid size, this residue was also replaced with the larger amino acid Thr (S389T) with the aim of narrowing the space. The two PhaC_Ac_ mutants with NSDG mutations and either S389A/T mutations were generated by site-directed mutagenesis and evaluated for P(3HB-*co*-3HHx) biosynthesis. The results are presented in Table 3. The additional S389A mutation did not alter the 3HHx fraction. However, the S389T mutation in PhaC_Ac_ NSDG increased the 3HHx fraction to 14.9 mol% without a significant decrease in PHA yield. Since the 3HHx fraction increased due to replacement with the bulkier amino acid in the mutant, further replacements were conducted using Val, Leu, Ile, and Cys, which have bulkier side chains than Ser based on their van der Waals volumes (Darby and Creighton, 1995; Tsuge et al., 2009). As a result, a slight increase in the 3HHx fraction up to 13.8 mol% was observed by introducing S389V/L/I/C mutations in PhaC_Ac_ NSDG. Of the mutations tested, the S389T mutation was the most effective in increasing the 3HHx fraction, followed by S389V. It was found that mutagenesis at position 318 in PhaC_Ac_ may enhance the 3HHx polymerization ability, although replacement with relatively bulky amino acids was effective.

**TABLE 3:** Polyhydroxyalkanoate accumulation in *Ralstonia eutropha* strain 005dC1Z126TRCB by expressing PhaC_Ac_ NSDG with S389X mutation.

**Expressed PhaC_Ac_**	**Dry cell wt. (g/L)**	**PHA content (wt.%)**	**PHA concentration (g/L)**	**Residual cell mass (g/L)**	**3HHx (mol%)**	**3HO (mol%)**	**Size increment (Å^3^)^a^**
NSDG	17.8 ± 0.3	85.7 ± 0.8	15.2 ± 0.3	2.5 ± 0.1	13.1 ± 0.1	trace	0
NSDG/S389A	17.5 ± 0.3	84.3 ± 0.3	14.6 ± 0.0	2.7 ± 0.1	13.1 ± 0.1	trace	−6
NSDG/S389T	16.9 ± 0.2	83.7 ± 1.4	14.1 ± 0.4	2.7 ± 0.2	14.9 ± 0.1	0.2 ± 0.1	20
NSDG/S389V	16.6 ± 0.3	84.2 ± 1.2	14.1 ± 0.5	2.8 ± 0.5	13.8 ± 0.2	trace	32
NSDG/S389L	17.8 ± 0.5	86.7 ± 1.1	15.4 ± 0.2	2.4 ± 0.4	13.5 ± 0.1	trace	51
NSDG/S389I	17.4 ± 0.3	78.7 ± 3.0	13.7 ± 0.1	3.7 ± 0.6	13.7 ± 0.1	trace	51
NSDG/S389C	17.6 ± 0.2	82.8 ± 0.3	14.6 ± 0.2	3.0 ± 0.1	13.7 ± 0.0	trace	13

*Cells were cultured in mineral salt medium containing 1.5 w/v% palm kernel oil at 30 °C for 72 h.*

*Results are mean ± standard deviations from three separate experiments.*

*3HHx: 3-hydroxyhexanoate, 3HO: 3-hydroxyoctanoate, PHA: Polyhydroxyalkanoate, trace: less than 0.1 mol%.*

*^a^Size increment at position 389 from the wild-type PhaC_Ac_.*

### PHA Synthesis by PhaC_Ac_ NSDG With Additional Mutation for L436

L436 is an amino acid located slightly outside the active center, which corresponds to Y440 in PhaC_Re_. As predicted by homology modeling, the cavity of PhaC_Ac_ is larger than that of PhaC_Re_ because of the difference in the amino acid side size at this position. To examine the effect of mutagenesis for L436 on the 3HHx polymerization ability of PhaC_Ac_ NSDG, site-directed mutagenesis was performed. To examine the expanding effect of the pocket space, L436A/V mutations were introduced into PhaC_Ac_ NSDG. In addition, L436Y/I mutations were introduced to examine the opposite narrowing effect (Darby and Creighton, 1995; Tsuge et al., 2009). The results are listed in Table 4. PHA accumulation was observed for all strains with polymer contents greater than 80 wt%. However, these mutations showed a decrease in the 3HHx fraction; The L436A and L436Y mutations showed 21% and 66% reductions in the 3HHx fraction, respectively, when compared to the parental NSDG strain. Based on this observation, the residue at position 436 may be involved in substrate recognition, but mutagenesis at this position did not result in an increase in the 3HHx fraction of the polymer.

**TABLE 4:** Polyhydroxyalkanoate accumulation in *Ralstonia eutropha* strain 005dC1Z126TRCB by expressing PhaC_Ac_ NSDG with L436X mutation.

**Expressed PhaC_Ac_**	**Dry cell wt. (g/L)**	**PHA content (wt.%)**	**PHA concentration (g/L)**	**Residual cell mass (g/L)**	**3HHx (mol%)**	**3HO (mol%)**	**Size increment (Å^3^)^a^**
NSDG	17.8 ± 0.3	85.7 ± 0.8	15.2 ± 0.3	2.5 ± 0.1	13.1 ± 0.1	trace	0
NSDG/L436A	18.0 ± 0.2	81.4 ± 0.7	14.6 ± 0.1	3.5 ± 0.3	10.3 ± 0.2	0	−57
NSDG/L436V	17.6 ± 0.4	84.5 ± 0.5	15.1 ± 0.1	2.8 ± 0.1	13.0 ± 0.2	trace	−19
NSDG/L436Y	17.3 ± 0.1	80.6 ± 1.7	13.9 ± 0.3	3.3 ± 0.1	4.5 ± 0.1	0	17
NSDG/L436I	18.4 ± 0.3	85.1 ± 1.6	15.7 ± 0.5	2.7 ± 0.2	12.9 ± 0.3	0	0

*Cells were cultured in mineral salt medium containing 1.5 w/v% palm kernel oil at 30 °C for 72 h.*

*Results are mean ± standard deviations from three separate experiments.*

*3HHx: 3-hydroxyhexanoate, 3HO: 3-hydroxyoctanoate, PHA: Polyhydroxyalkanoate, trace: less than 0.1 mol%.*

*^a^Size increment at position 436 from the wild-type PhaC_Ac_.*

### Kinetic Analysis of PhaC_Ac_ NSDG With S389V/T/C Mutations

To obtain a better understanding of the polymerization ability of the 3HHx monomer of PhaC_Ac_, granule-associated PHA synthases were prepared and used for enzyme kinetic analysis. The granule-associated PHA synthase does not exhibit a lag phase (Gerngross et al., 1994; Taguchi et al., 2002) because the enzyme is already activated and thus is suitable for use in accurate kinetic analysis. To determine the PhaC_Ac_ concentrations on the surface of the isolated PHA granules, western blotting was performed using an antibody against PhaC_Ac_. The kinetic parameters determined for wild-type PhaC_Ac_, NSDG mutant, and NSDG/S389X mutants are listed in Table 5. The NSDG mutant and NSDG/S389X showed a lower Michaelis constant (*K*_*m*_) for the *R*-3HHx-CoA substrate than the wild-type PhaC_Ac_ but was not significant for the *R*-3HB-CoA substrate. In addition, the NSDG mutant and NSDG/S389X mutants showed a higher turnover number (*k*_*cat*_) for both substrates than the wild-type PhaC_Ac_, except for NSDG/S389V toward *R*-3HHx-CoA. Kinetic analysis revealed that the substrate affinity and turnover number, especially for *R*-3HHx-CoA, increased in the NSDG mutant. Among the mutants tested, the *K*_*m*_ values of S389V/C mutants for *R*-3HHx-CoA, which were 0.46 mM and 0.53 mM, respectively, showed smaller values than that of the parental NSDG strain (0.73 mM). The decrease in *K*_*m*_ value indicates the increased affinity between enzyme and substrate, thus providing evidence of the reinforced ability of 3HHx polymerization by these mutations. In contrast, by introducing the S389T mutation into PhaC_Ac_ NSDG, the *K*_*m*_ value slightly increased for both *R*-3HB-CoA and *R*-3HHx-CoA. Furthermore, the *k*_*cat*_ value significantly increased for both substrates by up to 3.4-fold compared to the parental NSDG enzyme. Thus, the increase in the 3HHx fraction caused by the S389T mutation could be attributed to the increased catalytic turnover of the enzyme, rather than the increased affinity between the substrate and the enzyme.

**TABLE 5:** Kinetic parameters of PHA granule-associated PhaC_Ac_ for *R*-3HB-CoA and *R*-3HHx-CoA substrates.

**PhaC_Ac_ enzyme**	***R*-3HB-CoA**		***R*-3HHx-CoA**	
	***K*_*m*_ (mM)^a^**	***k*_*cat*_ (min^–1^)^b^**	***K*_*m*_ (mM)^a^**	***k*_*cat*_ (min^–1^)^b^**
Wild type	1.23	260	1.05	153
NSDG	1.26	599	0.73	259
NSDG/S389V	1.05	385	0.46	130
NSDG/S389T	1.34	1913	0.79	876
NSGD/S389C	1.30	898	0.53	304

*^a^Michaelis constant.*

*^b^Turnover number calculated as one catalytic site in a dimerized PhaC_Ac_.*

*PHA, Polyhydroxyalkanoate.*

## Discussion

This study aimed to increase the 3HHx fraction in P(3HB-*co*-3HHx) by engineering PhaC_Ac_. Based on evolutionary engineering, we had already generated a PhaC_Ac_ NSDG mutant as a workhorse to synthesize a high 3HHx-fraction polymer. The mutation positions of NSDG are at the N-terminal region of PhaC_Ac_, and these amino acid residues are predicted to not be involved in the formation of the substrate pocket. Thus, to further modify the PhaC_Ac_ NSDG for higher 3HHx-fraction polymer synthesis, we attempted to change the cavity space of the substrate pocket by replacing certain amino acids. Recently, two research groups have published the partial crystal structure of PhaC_Re_ (Wittenborn et al., 2016; Kim et al., 2017). PhaC_Re_ can polymerize up to C5 monomers, whereas PhaC_Ac_ is capable of polymerizing up to C6 monomers. The difference in substrate specificity may be caused by the size of the substrate pocket near the active center (Kim et al., 2017). From this viewpoint, the three-dimensional structures around the cavity pocket space of PhaC_Re_ and the homology model of PhaC_Ac_ were compared, mainly focusing on the difference in the spread of amino acid side chains. As possible determining residues for the pocket size of PhaC_Ac_, three amino acids, namely Y318, S389, and L436, were identified in this study.

Our homology model suggests that Y318 may be an important residue that determines the pocket size (Figure 2B). Interestingly, this position is Ala in PHA synthases from *Pseudomonas* spp. (class II) that can polymerize MCL monomers up to C14. Therefore, it is reasonable to hypothesize that a mutation at this position has a significant influence on the pocket depth. The amino acid at this position in PhaC_Re_ (F318) has been suggested to stabilize the structure of the substrate pocket (Kim et al., 2017). Indeed, mutagenesis at this position of PhaC_Re_ led to a decrease in 75% of the synthase activity (Kim et al., 2017). In our study, mutation of Y318 of PhaC_Ac_ also resulted in a significant reduction in polymer synthesis (Table 2). Y318 maintains the structure of the substrate pocket and is strongly related to the polymerization ability in the same manner as PhaC_Re_.

The docking simulation using the crystal structure of PhaC_Re_ suggested that Y440 is located in the substrate entrance tunnel and contributes to the structural stabilization of the β-mercaptoethylamine/pantothenate (β-MP) moiety of *R*-3HB-CoA (Kim et al., 2017). Y440 stabilizes the substrate orientation by interacting with neighboring amino acids to efficiently catalyze the polymerization reaction. In PhaC_Ac_, the corresponding L436 was considered to regulate the space of the substrate entrance tunnel based on the homology model (Figure 2D). In fact, mutagenesis of L436 limited the substrate specificity of PhaC_Ac_ and reduced the 3HHx fraction in the biosynthesized polymer (Table 4). Among the NSDG/L436X mutants examined, the most remarkable reduction in the 3HHx fraction was observed for the NSDG/L436Y mutant, probably due to the narrowest pocket space by replacement with the largest amino acid Tyr.

However, the effect of 3HHx polymerization ability cannot always be explained by the reduction and expansion of pocket space due to amino acid replacement. In this study, we found that the 3HHx fraction in PHA increased after narrowing the substrate pocket by mutagenesis of S389 (Table 3). However, this observation was opposite to our hypothesis.

To better understand the effect of S389 mutagenesis, the kinetics of the enzymes with the S389X mutation were investigated. Kinetic analysis provided new information on the changes in catalytic function due to S389X mutations. It was revealed that substrate affinity for *R*-3HHx-CoA was increased by S389V/C mutations, whereas the catalytic turnover of the enzyme was increased by the S389T mutation. Thus, the increase in the 3HHx fraction caused by the S389T mutation may be due to the increased catalytic turnover of the enzyme, rather than the change in binding affinity between the enzyme and substrate. The relationship between pocket size narrowing and 3HHx polymerization ability may be explained by stabilization of the substrate orientation when the substrate accesses the active site. The proper orientation of the substrate may increase the efficiency of the catalytic reaction. However, further studies are required to elucidate the underlying mechanisms of mutagenesis.

## Conclusion

In conclusion, by comparing the substrate pocket structures of PhaC_Re_ and PhaC_Ac_, a new beneficial mutation position at S389 was found to enhance the 3HHx polymerization ability of PhaC_Ac_ NSDG. Since the discovery of the NSDG mutation, additional mutations conferring a superior ability of 3HHx polymerization have not been found by an evolutionary engineering approach. Thus, this is a successful example of PHA synthase engineering by effectively exploiting the findings from the three-dimensional structure of proteins.

## Data Availability Statement

The raw data supporting the conclusion of this article will be made available by the authors, without undue reservation.

## Author Contributions

KH, SK, KO, SY, TT, and SS jointly conceived the study. KH, SK, KO, and SY performed the experiments. KH wrote the manuscript in consultation with SK, KO, SY, TT, and SS. All authors read and approved the final manuscript.

## Conflict of Interest

KH, SK, KO, SY, and SS are employees of Kaneka, Co., Ltd. The remaining author declares that the research was conducted in the absence of any commercial or financial relationships that could be construed as a potential conflict of interest.

## Footnotes

1 http://rsbweb.nih.gov/ij/
