# Supplementary material for: Engineering of Aeromonas caviae Polyhydroxyalkanoate Synthase Through Site-Directed Mutagenesis for Enhanced Polymerization of the 3-Hydroxyhexanoate Unit
Source: Front Bioeng Biotechnol. 2021 Mar 3;9:627082. doi: 10.3389/fbioe.2021.627082 (PMC7966705; doi:10.3389/fbioe.2021.627082)
Supplement: Supplementary file 1 [file Table_1.DOCX]

Supplementary Materials for

**Engineering of *Aeromonas caviae* Polyhydroxyalkanoate Synthase through Site-Directed Mutagenesis for Enhanced Polymerization of the 3-Hydroxyhexanoate Unit**

Ken Harada, Shingo Kobayashi, Kanji Oshima, Shinichi Yoshida, Takeharu Tsuge, Shunsuke Sato


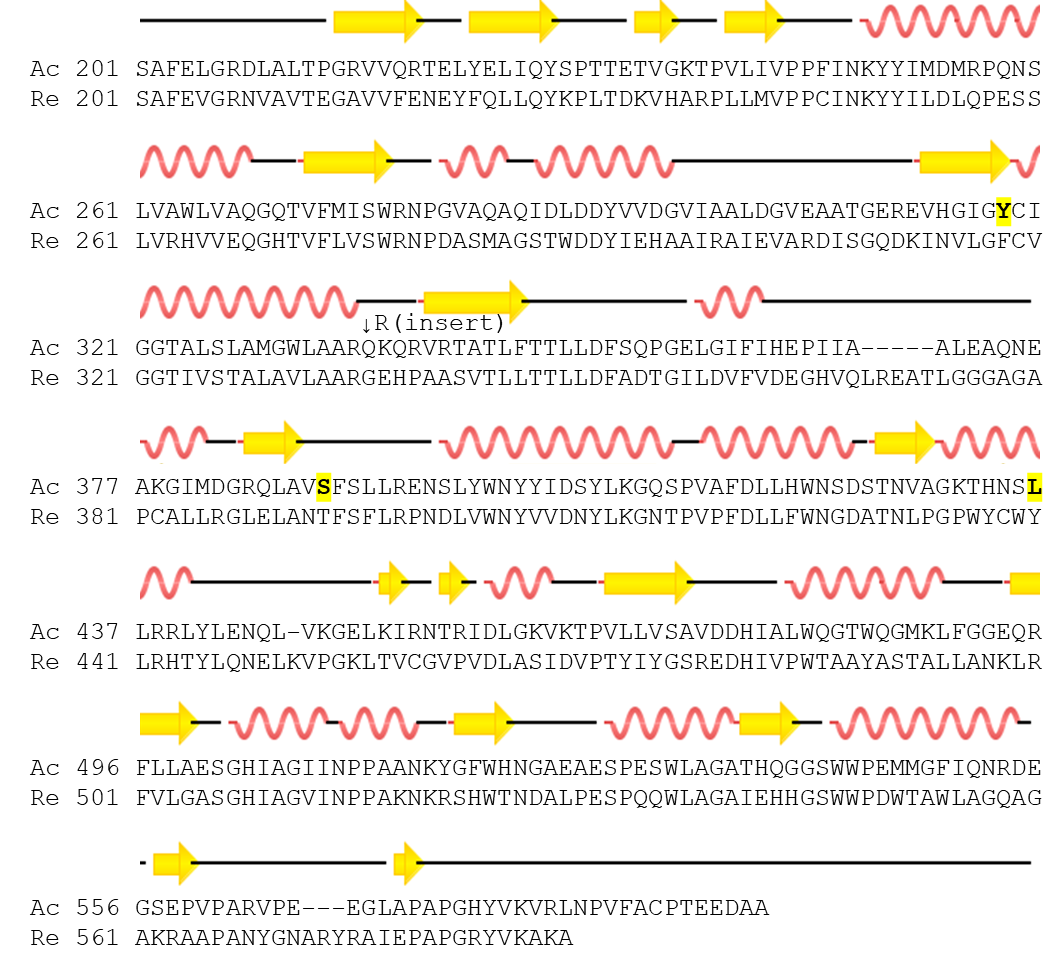


**Supplementary Figure 1**. Sequence alignment of PhaC_Ac_ and PhaC_Re_. Upper sequence is PhaC_Ac_ and lower sequence is phaC_Re_ (amino acid sequence 201-589). Secondary structure is schematically shown. α helix is shown by red and β strand is shown by yellow arrow.

| **Table S1** Primers used in this study | |
| --- | --- |
| Primers | Sequence |
| MunI_PhaCAc_F | CAATTGCACGTGCAGAGAGACAATCAAATCATGAGCCAACCATCTTATGG |
| SpeI_PhaCAc_R | ACTAGTCGGCTGCCGACTGGTTGAACCAGGCCGGCAGGTCATGCGGCGTCCTCCTCTGTT |
| pCUP3_IF_MunI_trp_F | **ACATTGCGCTGAAAGAAGGGC**CAATTGTGCTTCTGGCGTC |
| pCUP3_SpeI_IF_R | **GCTCGGATCC**ACTAGTCGGCTGCCGACTGGT |
| phaCAc_Y318X_F | TGCATCGGCGGCACCGCCCT |
| phaCAc_Y318M_R | AGGGCGGTGCCGCCGATGCACATGCCGATGCCGTGCACCTCCCG |
| phaCAc_Y318I_R | AGGGCGGTGCCGCCGATGCAGATGCCGATGCCGTGCACCTCCCG |
| phaCAc_Y318L_R | AGGGCGGTGCCGCCGATGCACAGGCCGATGCCGTGCACCTCCCG |
| phaCAc_L436X_F | CTGCGCCGTCTCTACCTGGAGAAC |
| phaCAc_L436A_R | TCTCCAGGTAGAGACGGCGCAGCGCGCTGTTGTGGGTCTTGCCCGC |
| phaCAc_L436V_R | TCTCCAGGTAGAGACGGCGCAGCACGCTGTTGTGGGTCTTGCCCGC |
| phaCAc_L436Y_R | TCTCCAGGTAGAGACGGCGCAGGTAGCTGTTGTGGGTCTTGCCCGC |
| phaCAc_L436I_R | TCTCCAGGTAGAGACGGCGCAGGATGCTGTTGTGGGTCTTGCCCGC |
| phaCAc_S389X_F | TTCAGCCTGCTGCGGGAGAACAG |
| phaCAc_S389A_R | CTGTTCTCCCGCAGCAGGCTGAACGCGACCGCCAGCTGGCGCCCGT |
| phaCAc_S389T_R | CTGTTCTCCCGCAGCAGGCTGAAAGTGACCGCCAGCTGGCGCCCGT |
| phaCAc_S389V_R | CTGTTCTCCCGCAGCAGGCTGAACACGACCGCCAGCTGGCGCCCGT |
| phaCAc_S389L_R | CTGTTCTCCCGCAGCAGGCTGAACAGGACCGCCAGCTGGCGCCCGT |
| phaCAc_S389I_R | CTGTTCTCCCGCAGCAGGCTGAAGATGACCGCCAGCTGGCGCCCGT |
| phaCAc_S389C_R | CTGTTCTCCCGCAGCAGGCTGAAGCAGACCGCCAGCTGGCGCCCGT |
| The underlined sequences indicate restriction enzyme sites or mutated sites. The bold sequences indicate In-Fusion alignment. | |
